# Α Humanized RANKL Transgenic Mouse Model of Progestin-Induced Mammary Carcinogenesis for Evaluation of Novel Therapeutics

**DOI:** 10.3390/cancers15154006

**Published:** 2023-08-07

**Authors:** Anthi Kolokotroni, Evi Gkikopoulou, Vagelis Rinotas, Lydia Ntari, Danae Zareifi, Maritina Rouchota, Sophia Sarpaki, Ilias Lymperopoulos, Leonidas G. Alexopoulos, George Loudos, Maria C. Denis, Niki Karagianni, Eleni Douni

**Affiliations:** 1 Laboratory of Genetics, Department of Biotechnology, Agricultural University of Athens, Iera Odos 75, 11855 Athens, Greece; 2 Institute for Bioinnovation, Biomedical Sciences Research Center “Alexander Fleming”, Fleming 34, 16672 Vari, Greece; 3 Biomedcode Hellas SA, Fleming 34, 16672 Vari, Greecemdenis@biomedcode.com (M.C.D.);; 4 Department of Mechanical Engineering, National Technical University of Athens, 10682 Athens, Greece; 5 BIOEMTECH, Lefkippos Attica Technology Park, NCSR “Demokritos”, Ag. Paraskevi, 15343 Athens, Greecegeorge@bioemtech.com (G.L.); 6 1st Breast Clinic, Iaso Hospital, 37-39 Kifissias, 15123 Marousi, Greece

**Keywords:** RANKL, mammary carcinogenesis, breast cancer model, hormone-driven breast cancer mouse model, MPA/DMBA-induced tumors, Denosumab, preclinical studies

## Abstract

**Simple Summary:**

Targeting receptor activator of nuclear factor-κB ligand (RANKL) with the monoclonal antibody Denosumab decreases osteoclast-mediated bone resorption and is approved for the treatment of postmenopausal osteoporosis. Since RANKL is also implicated in mammary gland homeostasis and breast tumorigenesis, Denosumab is being currently pursued as a candidate for drug repurposing in oncology, including breast cancer, while its efficacy remains controversial. In this study, by developing a humanized transgenic mouse model of human RANKL overexpression, we demonstrated that RANKL mediated hormone-induced mammary carcinogenesis, while its prophylactic inhibition by Denosumab prevented tumorigenesis. Our humanized transgenic mice provide a unique genetic tool for investigating the involvement of human RANKL in breast cancer pathogenesis and can serve as a preclinical platform for anticancer therapies.

**Abstract:**

Receptor activator of nuclear factor-κB ligand (RANKL) is critically involved in mammary gland pathophysiology, while its pharmaceutical inhibition is being currently investigated in breast cancer. Herein, we investigated whether the overexpression of human RANKL in transgenic mice affects hormone-induced mammary carcinogenesis, and evaluated the efficacy of anti-RANKL treatments, such as OPG-Fc targeting both human and mouse RANKL or Denosumab against human RANKL. We established novel MPA/DMBA-driven mammary carcinogenesis models in TgRANKL mice that express both human and mouse RANKL, as well as in humanized humTgRANKL mice expressing only human RANKL, and compared them to MPA/DMBA-treated wild-type (WT) mice. Our results show that TgRANKL and WT mice have similar levels of susceptibility to mammary carcinogenesis, while OPG-Fc treatment restored mammary ductal density, and prevented ductal branching and the formation of neoplastic foci in both genotypes. humTgRANKL mice also developed MPA/DMBA-induced tumors with similar incidence and burden to those of WT and TgRANKL mice. The prophylactic treatment of humTgRANKL mice with Denosumab significantly prevented the rate of appearance of mammary tumors from 86.7% to 15.4% and the early stages of carcinogenesis, whereas therapeutic treatment did not lead to any significant attenuation of tumor incidence or tumor burden compared to control mice, suggesting the importance of RANKL primarily in the initial stages of tumorigenesis. Overall, we provide unique genetic tools for investigating the involvement of RANKL in breast carcinogenesis, and allow the preclinical evaluation of novel therapeutics that target hormone-related breast cancers.

## 1. Introduction

Breast cancer is considered a global disease since one out of eight women will develop this form of cancer during their lifetime. Even though survival rates of patients have been improved in the last few decades due to more effective therapies and mostly due to early diagnosis, breast cancer still remains the second leading cause of cancer-related death in women [1]. Breast cancer development has been associated with continuous hormone exposure, mostly progesterone and estrogen, during the luteal phase of the menstrual cycle that is characterized by intense epithelial proliferation [2,3]. A significant increase in breast cancer risk has been reported for women receiving estrogen plus synthetic progesterone (progestins) hormone replacement therapy (combined HRT) as compared with women treated with estrogen-only HRT [4,5]. In particular, the use of medroxyprogesterone acetate (MPA) in HRT and contraceptives has been shown to increase the risk of developing breast cancer [6,7].

Receptor activator of nuclear factor-κB ligand (RANKL) is a member of the tumor necrosis factor (TNF) superfamily and constitutes a key regulator in osteoclast development [8,9], inducing physiological and pathological bone resorption in osteolytic diseases, such as osteoporosis and arthritis [10]. RANKL functions through its cognate receptor RANK, and can be inhibited by the decoy soluble receptor osteoprotegerin (OPG). Denosumab, a human monoclonal antibody targeting human RANKL, has been approved for the treatment of postmenopausal osteoporosis since 2010 [11]. In addition to its role in bone resorption, RANKL also plays an essential role in the dynamic morphogenesis of the mammary gland, acting as a paracrine mediator of progesterone for the expansion of mammary progenitor cells during the physiological estrous cycle [12,13]. RANKL is produced by luminal cells that express progesterone receptor (PR) and signals to mammary stem (MaSC) and/or luminal progenitor subsets that express RANK [14]. In a similar manner, RANKL also drives the proliferation of mammary progenitors required for the development of lobulo-alveolar structures during the course of pregnancy [15]. Thus, RANKL-deficient mice fail to form lobulo-alveolar structures during pregnancy, resulting in the death of newborns [15].

RANKL has also been implicated in breast tumorigenesis, while RANKL expression has been confirmed in human breast cancer specimens [16]. RANKL has been implicated in the development of progestin-induced breast cancer in mice, through a mechanism similar to the physiological progesterone-mediated epithelial cell proliferation occurring in the mammary gland [17,18]. More specifically, the treatment of female mice with MPA resulted in the profound upregulation of RANKL expression in the mammary epithelium, leading to increased epithelial cell proliferation, which was abolished in RANK-deficient mice [17]. Similarly, the induction of breast cancer by the co-administration of MPA and the carcinogenic agent 7,14-dimethylbenz[a]anthracene (DMBA) in RANK-deficient mice resulted in the delayed onset of mammary tumor development and increased survival rates [17]. On the other hand, RANK overexpression in the mammary gland of transgenic mice resulted in accelerated pre-neoplasia and tumors following MPA/DMBA treatment, an effect that was attenuated with the pharmacological inhibition of RANKL [19].

In this study, we have developed novel preclinical tools and provided further evidence that supports the potential therapeutic effect of established anti-RANKL agents (OPG-Fc, Denosumab) in attenuating the development of progesterone-dependent and RANKL-mediated mammary carcinogenesis. More specifically, we induced hormone-dependent (MPA/DMBA) breast carcinogenesis in transgenic mice expressing human RANKL (huRANKL) [20], and established a pre-clinical platform to evaluate the efficacy of therapeutics targeting huRANKL. Our work provides evidence that supports the beneficial effects of RANKL inhibition in breast cancer tumorigenesis.

## 2. Materials and Methods

### 2.1. Mouse Husbandry

TgRANKL (Tg5519 line) [20], humTgRANKL (Tg5519/RANKL^tles/tles^) [21], and WT control female mice (all in C57BL/6 background) were maintained and bred in the animal facility of Biomedical Sciences Research Center (B.S.R.C.) “Alexander Fleming” (registered code EL09BIO05) under specific pathogen-free conditions. All animal work was approved by the Institutional Protocol Evaluation Committee and was licensed by the Veterinary Authorities of Attica Prefecture (registered codes: 533/13 February 2019 and 105612/6 February 2020) in compliance with the animal welfare guidelines of the PD 56/2013 and the European Directive 2010/63/EU.

### 2.2. MPA/DMBA-Induced Mammary Carcinogenesis

**Figure 1:**
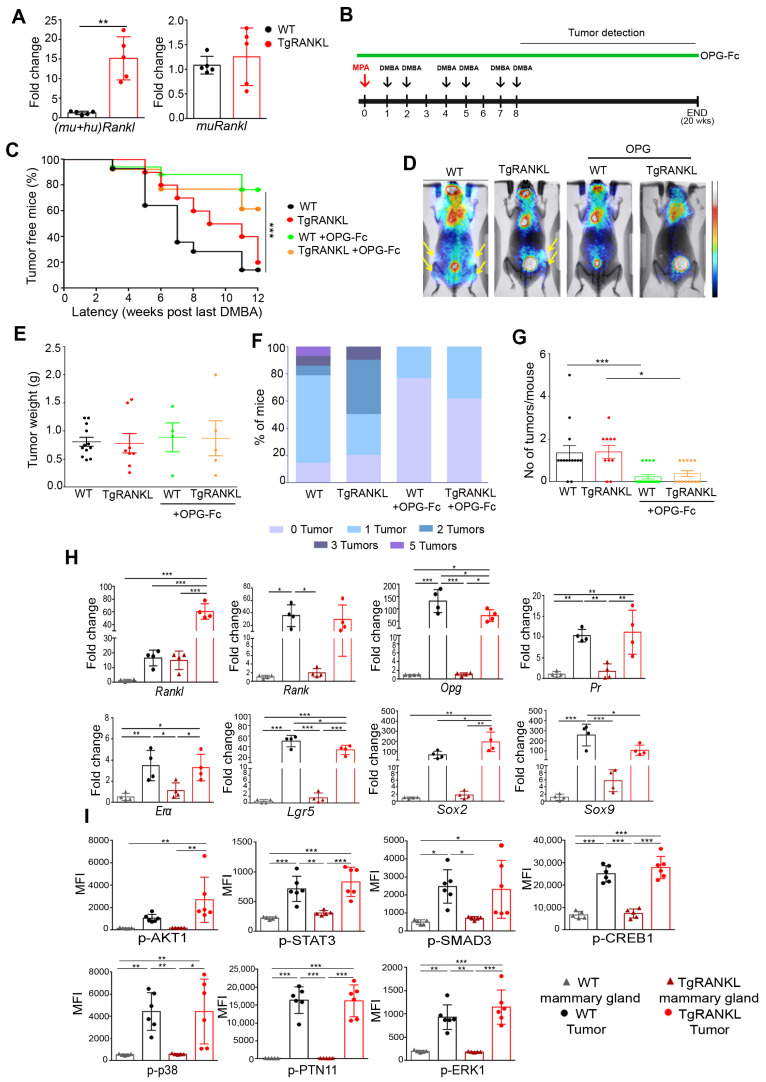
Comparison of MPA/DBMA-induced mammary tumorigenesis between WT and TgRANKL mice. (**A**) Comparative expression analysis for total *Rankl* (*mu + hu*) or only for endogenous *muRankl* in the mammary glands of WT and TgRANKL mice (n = 5/group). Statistical analysis was performed with Mann–Whitney unpaired *t*-test. (**B**) Carcinogenesis scheme induced by MPA and DMBA. Six-week-old female mice were implanted subcutaneously with MPA pellets and orally received 6 doses of DMBA during a period of eight weeks. OPG-Fc was administered twice weekly for the whole duration of the experiment, starting one day before MPA implantation. (**C**) Onset kinetics of palpable MPA/DMBA-induced mammary tumors in WT, TgRANKL either with or without treatment with OPG-Fc (n = 10–17/group). Percentage of tumor-free mice is presented for the period after the last DMBA challenge to the end of the experiment. (**D**) Representative images from planar PET scanning. Yellow arrows indicate tumor signal. (**E**) Tumor weight, (**F**) % of mice bearing from 0 to 5 tumors per experimental group and (**G**) number of tumors per mouse in each experimental group. Comparison in G was performed between WT untreated and WT treated with OPG-Fc as well as between TgRANKL untreated and TgRANKL treated with OPG-Fc using Mann–Whitney unpaired t-test (* *p* < 0.05, *** *p* < 0.001). (**H**) Comparative expression analysis for *Rankl(mu + hu)*, *Rank*, *Opg*, *Pr*, *Erα*, *Lgr5*, *Sox2*, and *Sox9* in mammary glands and tumors from WT and TgRANKL mice (n = 4/group). (**I**) Multiplex ELISA analysis for phospho-proteins p-AKT1, p-STAT3, p-SMAD3, p-CREB1, p-p38, p-PTN11 and p-ERK1 in mammary glands and tumors from WT and TgRANKL mice (n = 5–6/group). Comparison was performed with one-way ANOVA and Τukey’s post hoc test (* *p* < 0.05, ** *p* < 0.01, *** *p* < 0.001).

Treatment with MPA and DMBA was performed as previously described [17]. Briefly, 6-week-old female mice were subcutaneously implanted in the upper back area with 90-day slow-release pellets containing 50 mg medroxyprogesterone acetate (MPA) (Innovative Research of America). In total, 200 μL of a 5 mg/mL solution of DMBA (Sigma-Aldrich, St Louis, MO, USA) in cottonseed oil was administered by oral gavage according to the scheme shown in Figure 1B. Mammary tumors were detected by palpation and mice were sacrificed either when tumor size exceeded 10–15 mm in diameter or when the overall condition of the mice fell in humane endpoint criteria.

### 2.3. Administration of OPG-Fc and Denosumab

TgRANKL and WT mice were treated twice weekly either intraperitoneally with 10 mg/kg OPG-Fc (supplied by Amgen Inc., Thousand Oaks, CA, USA) or subcutaneously with 10 mg/kg Denosumab (Amgen Inc.) until the end of the protocol. Prophylactic treatment was initiated one day before MPA implantation, while treatment was initiated on the last day of DMBA administration.

### 2.4. In Vivo Imaging

In vivo imaging for the detection of tumors was performed using the β-eye benchtop system with Positron Emission Tomography (PET) isotopes with an artificial intelligence-based X-ray system (ΒIOEMTECH, Athens, Greece). The tracer of choice was 2-deoxy-2-[^18^F]fluoro-D-glucose [^18^F]FDG, the gold standard for tumor investigation, which is also used in routine clinical practice [22]. Animals were anesthetized with isofluorane (3–5% for induction and 1–3% for maintenance) and were kept warm during all imaging scans. In total, 25–35 uCi/100 μL of [^18^F]FDG [23] was administered intravenously via the retro-orbital vein. Dynamic screening was performed for the first hour post-injection, while 10 min-long static imaging was performed at later time points (2 and 3 h post-injection), to identify the best time point at which maximum tumor uptake would be observed. The 2 h post-injection point was selected as the optimal time point for screening such mammary tumors with [^18^F]FDG. Post-processing was performed using the accompanying eyes software tool (visual|*eyes*^TM^, Version 3, BIOEMTECH, Athens, Greece) and smoothing using a Gaussian filter (1.4 mm, isotropic).

### 2.5. Whole-Mount Analysis, Carmine-Alum Staining of Mammary Glands

Mammary glands and tumors were isolated and fixed overnight at 4 °C with 4% paraformaldehyde (PFA) in phosphate-buffered saline (PBS). The preparation and whole-mount staining with carmine-alum was performed as previously described [24].

### 2.6. Histology and Immunohistochemistry

Mammary glands and tumors were fixed overnight at 4 °C with 4% PFA in PBS. For histological analysis, the tissues were embedded in paraffin and 5 μm tissue sections were obtained. Tissue sections were stained with hematoxylin and eosin (H/E) and histology images were acquired using a Nikon E800 upright widefield/fluorescence microscope. For immunohistochemical staining (IHC), antigen retrieval was first performed by heating for 5 min at 120 °C in a sodium citrate buffer at pH 6, followed by blocking for 1 h at RT with 3% BSA in PBS and staining overnight at 4 °C with anti-Cytokeratin 8 at 1:250 (rabbit, Abcam, Cambridge, UK, ab53280) and anti-Cytokeratin 5 at 1:250 (rabbit, Abcam, ab52635). Endogenous peroxidase activity was inhibited by incubating with 3% hydrogen peroxide for 10 min. In a final step, sections were incubated with horseradish peroxidase (HRP)-conjugated anti-rabbit IgG (Santa Cruz Biotechnology, Dallas, TX, USA) for 1 h at RT, washed and treated with the substrate 3,3’-Diaminobenzidine (DAB) for 2–5 min in the dark. Hematoxylin was used for counterstaining.

### 2.7. MicroCT Tomography of Mammary Glands

Inguinal mammary glands were stained with phosphotungstic acid (PTA) to enhance imaging performed by microcomputed tomography (microCT) as previously described [24].

### 2.8. MicroCT Tomography Analysis of Femurs

Femurs were fixed overnight in 10% formalin, washed with water and stored in PBS. The microarchitecture of the distal femurs from all examined mice was evaluated through microCT (SkyScan1172, Bruker, Billerica, MA, USA). Images were acquired at 50 KV, 100 µA with a 0.5 mm aluminum filter and 6 μm voxel size. Three-dimensional reconstructions (8.8 mm cubic resolution) were generated using NRecon software, version 1.7.4.6 (Bruker) as previously described [25]. For the trabecular area of the distal femur, we assessed the bone volume fraction (BV/TV, %), the trabecular number (Tb.N, mm^−1^) and the trabecular separation (Tb.S, mm).

### 2.9. RNA Isolation and qPCR Analysis

Total RNA was extracted from tissues (TRI Reagent, MRC), and after DNase I treatment (Sigma-Aldrich), first-strand cDNA was synthesized using 2 μg of total RNA and M-MLV (Invitrogen, Carlsbad, CA, USA). Templates were amplified with HOT FIREPol^®^ EvaGreen Master Mix (Solis Biodyne) on the CFX96 Connect real-time PCR instrument (Bio-Rad Laboratories, Hercules, CA, USA). Data analysis was performed following the 2^−ΔΔCT^ method [26]. The primers were purchased from Eurofins Genomics and are listed in Table S1. The PCR was performed in a 20 μL reaction using 58 °C as the annealing temperature for 40 cycles. PCR using primers specific to the B2M gene was also carried out as an internal control, using the same cDNA samples and the same conditions. For each experiment at least four biological and two technical replicates were used.

### 2.10. Multiplex ELISA

Mammary tumors and mammary glands were ground to powder in liquid nitrogen using a pestle and mortar and were solubilized in 150 μL lysis buffer containing 100 mM Tris-HCl, pH 7.6, 4% SDS and freshly made 100 mM DTT, protease inhibitors cocktail (Protavio, Athens, Greece, PR-PIC), and phenylmethylsulfonyl fluoride 1M (PMSF, Sigma-Aldrich). Samples were homogenized, followed by 3-fold sonication in order to shear the DNA. Finally, the samples were centrifuged at 17,000× *g* for 15 min at 4 °C twice and the supernatants were transferred to new tubes. The total protein content of lysates was estimated (Pierce^TM^ BCA Protein Assay Kit, Thermo Scientific, Waltham, MA, USA) and absorbance was measured at 560 nm on a Varioskan LUX Multimode Microplate Reader (Thermo Scientific). The protein concentration was adjusted to 250 μg/mL or 300 μg/mL using Protavio lysis buffer (Protavio, Athens, Greece, PR-LYSB) prior to sample analysis. The samples were transferred into flat-bottom 96-well plates containing bead mix, pre-washed with Assay Buffer (Protavio, Athens, Greece, PR-ASSB pH 7.4) for multiplex ELISA experiments. Bead-based multiplex ELISA assays utilize color-coded microspheres to attain distinct fluorescent signatures for each assay combination. Antibodies recognising specific protein targets are coated on the bead surface, and a bead mix containing the different coated beads was used to analyze protein levels. Bead regions were uniquely identified and differentiated by the Luminex instrumentation based on their unique color classification [27].

A custom-developed 19-plex assay panel (10-plex: PR-PHOS-1-KIT, 9-plex PR-PHOS-2-KIT, Protavio, Athens, Greece) was used to determine the levels of selected phosphorylated proteins. Target protein assays and the phosphorylation residues detected are demonstrated in Table S2. Control cell lysates were prepared by Protavio (Athens, Greece) using several cell lines and stimuli to monitor the assay performance of the phospho-panels in each run. Signals were reported as Median Fluorescence Intensities (MFI) and samples with bead counts ≥ 20 for each bead region were included in the analysis. Multiplex ELISA assays were performed on a Luminex FLEXMAP 3D platform (Luminex, Austin, TX, USA).

### 2.11. Statistical Analysis

All results are presented as scatter dot-plots showing each data point as a mean value ± standard deviation (SD). Statistical significance was assessed by one-way analysis of variance (ANOVA) with the Tukey post-hoc test to compare means of multiple groups, while the *t*-test was used for the comparison of two groups. For all tests, *p* < 0.05 was considered statistically significant.

## 3. Results

### 3.1. Comparison of MPA/DMBA-Driven Mammary Carcinogenesis between Wild-Type Mice and Transgenic Mice Overexpressing Human RANKL

We have previously generated transgenic mice carrying a 200 kb genomic fragment containing both the coding and regulatory regions of the human *Rankl* (*huRankl*) gene [20]. These mice overexpress *huRankl* and spontaneously develop osteoporosis pathology. In the current study, we examined whether these mice also overexpress *huRankl* in their mammary glands, as it is known that in this tissue endogenous mouse, *Rankl* (*muRankl*) expression is induced by progesterone [28]. Using a pair of primers that can detect total *Rankl* (human and mouse), we found that the mammary glands of TgRANKL mice expressed significantly higher levels of *Rankl* compared to WT (Figure 1A). Since the mammary glands of TgRANKL and WT mice exhibit comparable expression levels of *muRankl* (Figure 1A), the increased expression of total *Rankl* in TgRANKL mammary glands is attributed to the high expression levels of *huRankl* transgene.

Then, we investigated whether TgRANKL mice were more susceptible than WT mice to hormone-induced mammary carcinogenesis. For this purpose, we established an MPA/DMBA carcinogenesis protocol that involved an 8-week-long treatment scheme with DMBA, starting just after the implantation of the MPA-releasing pellets with a total duration of 20 weeks (Figure 1B). The treatment of both WT and TgRANKL females with MPA/DMBA led to the development of mammary tumors that had similar tumor latency and progression (Figure 1C). Through in vivo PET scan imaging we managed to detect early-stage tumors in abdominal and inguinal mammary glands in MPA/DMBA-treated WT and TgRANKL mice (Figure 1D). No differences were observed between TgRANKL and WT mice in terms of tumor burden, including tumor weight, the percentage of mice bearing from zero to five tumors, and the number of tumors per mouse (Figure 1E–G).

Once the tumors reached a size of 10–15 mm in diameter, the mice were euthanized and the tumors as well as the adjacent mammary glands were harvested for histological and molecular analysis. Histopathological analysis of the MPA/DMBA-induced tumors confirmed the previously reported heterogeneity of tumors detected in this model [29], and highlighted as a prevalent tumor type the adenosquamous carcinoma with sporadic neoplastic glands and keratin pearls (Figure S1A). Tumors were primarily stained for the luminal marker cytokeratin 8 and, to a lesser extent, for the myoepithelial marker cytokeratin 5 (Figure S1B,C).

At the molecular level, the expressions of total *Rankl*, *Rank,* and *Opg* were found significantly increased in mammary tumors compared to mammary glands, both in WT and TgRANKL mice (Figure 1H). Tumors also displayed increased expression for genes encoding progesterone receptor (*Pr*), estrogen receptor α (*Erα*), and the stem cell markers *Lgr5*, *Sox2* and *Sox9* in both genotypes (Figure 1H). The phosphorylation analysis of signaling proteins through multiplex ELISA demonstrated increased phosphorylation in AKT1, STAT3, SMAD3, CREB1, p-38, PTN11, and ERK1 in tumors compared to mammary glands in both genotypes (Figure 1I and Figure S2). The above results demonstrate that TgRANKL mice overexpressing huRANKL in the mammary glands have similar levels of susceptibility to MPA/DBMA-induced mammary carcinogenesis with WT mice.

### 3.2. Treatment with OPG-Fc Prevents MPA/DMBA-Induced Mammary Carcinogenesis

To examine whether the MPA/DMBA-induced mammary carcinogenesis model is RANKL-dependent, we treated WT and TgRANKL mice with OPG-Fc, an inhibitor of both human and mouse RANKL [30,31,32]. Treatment with OPG-Fc was initiated one day before MPA implantation and continued throughout the study (Figure 1B). The efficacy of the OPG-Fc treatment was confirmed with the reversal of RANKL-mediated bone resorption in femurs through microCT analysis (Figure S3). OPG-Fc led also to an increase in the tumor latency both in WT and TgRANKL mice (Figure 1C). Ιn vivo imaging using planar PET scan detected tumors in WT and TgRANKL mice, but not in the groups treated with OPG-Fc (Figure 1D). Moreover, OPG-Fc treatment led to a reduction in the tumor burden both in WT and TgRANKL mice, as it prevented the formation of multiple tumors per mouse (Figure 1F,G). However, once formed, the tumors had similar weights and characteristics compared to mice that had not received OPG-Fc treatment (Figure 1E), suggesting the involvement of RANKL mainly at the early stages of mammary tumorigenesis.

**Figure 2:**
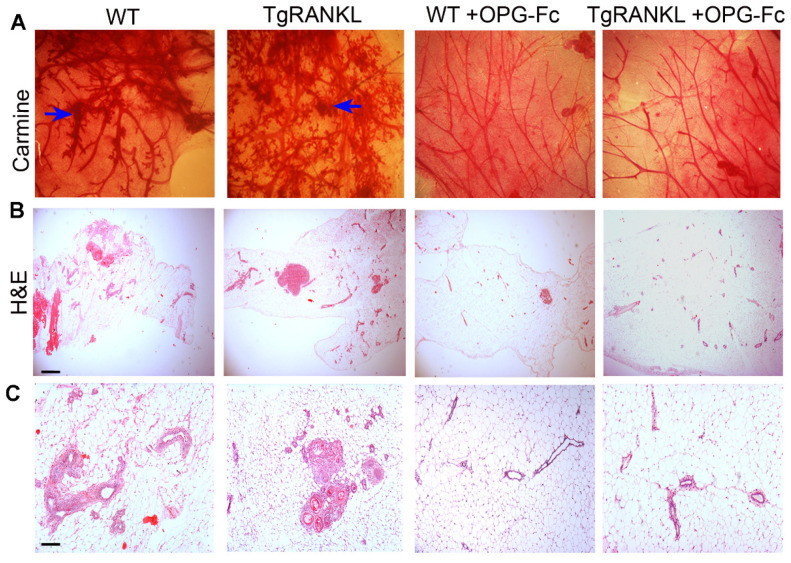
RANKL inhibition with OPG-Fc prevented side-branching and neoplastic epithelial foci in the mammary glands of MPA/DMBA-treated mice. Representative images of mammary glands adjacent to tumors from mice either treated with OPG-Fc or not, stained with (**A**) carmine alum and (**B**,**C**) hematoxylin and eosin. Blue arrows indicate mammary neoplastic foci (n = 5–8/group). Scale bars, 150 μm and 80 μm for H&E ((**B**) and (**C**), respectively).

We also investigated the morphology of the mammary glands adjacent to tumors in the presence of prophylactic OPG-Fc treatment. Whole-mount carmine alum staining and histological analysis revealed a dense epithelial ductal network with prominent side-branching and neoplastic epithelial foci in MPA/DMBA-treated WT and TgRANKL mice (Figure 2A–C). Treatment with OPG-Fc restored the mammary ductal density and prevented the formation of ductal branching and mammary neoplastic foci, maintaining the normal ductal tree structure (Figure 2A–C). Altogether, these results indicate that the MPA/DMBA-induced tumors are largely RANKL-dependent.

### 3.3. Generation of a Humanized RANKL Transgenic Mouse Model of Mammary Carcinogenesis

**Figure 3:**
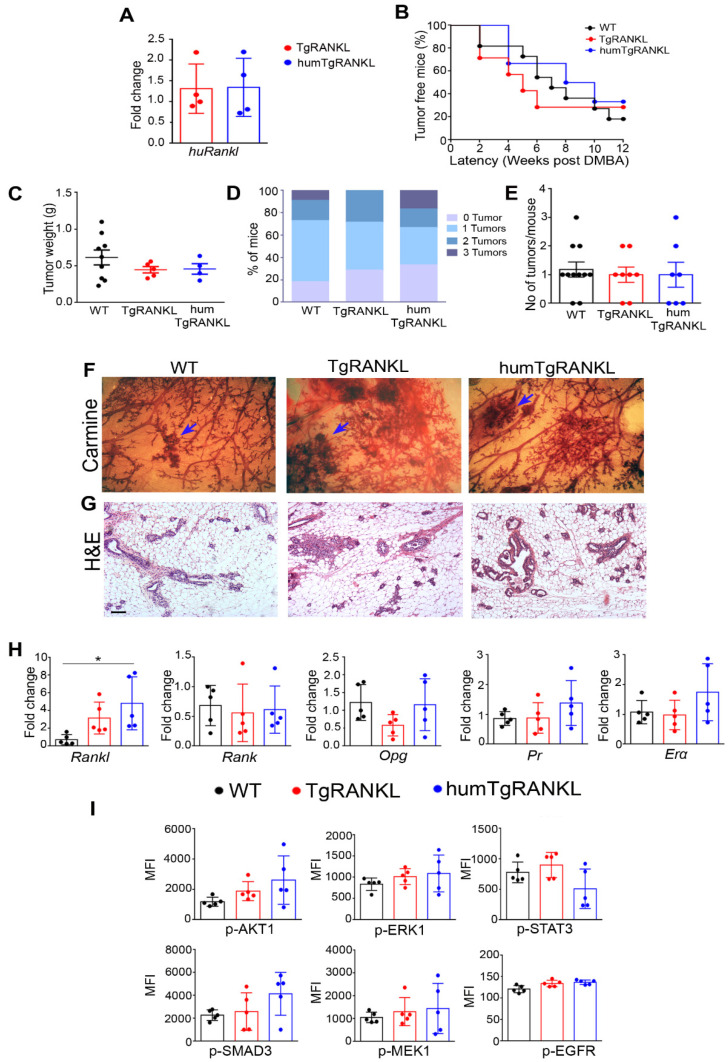
MPA/DMBA-induced mammary carcinogenesis in humTgRANKL mice. (**A**) Comparative expression analysis for *huRankl* in TgRANKL and humTgRANKL mammary glands (n = 4/group). Statistical analysis was performed with unpaired *t*-tests. (**B**) Onset kinetics of palpable MPA/DMBA-induced mammary tumors in WT, TgRANKL and humTgRANKL females (n = 7–10/group). (**C**) Tumor weight, (**D**) % of mice bearing from 0 to 3 tumors per experimental group, and (**E**) number of tumors per mouse in each experimental group. Adjacent mammary glands of WT, TgRANKL and humTgRANKL mice were stained with (**F**) carmine alum and (**G**) hematoxylin and eosin. Blue arrows indicate mammary neoplasia. Scale bar, 80 μm for histology. (**H**) Comparative expression analysis for *Rankl(mu+hu)*, *Rank*, *Opg*, *Pr* and *Erα* in MPA/DMBA-induced tumors from WT, TgRANKL and humTgRANKL mice (n = 5/group). (**I**) Multiplex ELISA analysis for phospho-proteins p-AKT1, p-ERK1, p-STAT3, p-SMAD3, p-MEK, and p-EGFR (n = 5/group). Data are shown as mean ± SD. One-way ANOVA and Τukey’s post hoc test were performed (* *p* < 0.05).

The induction of mammary carcinogenesis through the administration of MPA/DMBA in the TgRANKL mice includes the involvement of both mouse and human RANKL. To investigate whether the *huRankl* transgene can independently mediate hormone-induced mammary carcinogenesis, we generated a humanized (humTgRANKL) transgenic mouse model exclusively expressing *huRankl*. Humanized mice were generated by breeding the osteoporotic TgRANKL mice with the osteopetrotic RANKL^tles/tles^ mice carrying a missense mutation that abolishes the function of muRANKL [21]. qPCR analysis showed that the mammary glands of TgRANKL and humTgRANKL mice had comparable expression levels of *huRankl* (Figure 3A).

Interestingly, in humTgRANKL mice, the introduction of the *huRankl* transgene in the *muRankl*-deficient genetic background not only reversed the osteopetrotic phenotype of RANKL^tles/tles^ mice, but also allowed the development of an osteoporotic phenotype similar to the one observed in TgRANKL mice (Figure S4). Humanized mice developed MPA/DMBA-induced tumors with similar latency, weight, and number per mouse to those observed in WT and TgRANKL mice (Figure 3B–E). Whole-mount carmine alum staining and histological analysis of the mammary glands adjacent to tumors showed that humTgRANKL mice developed a ductal structure with high density, epithelial hyperplasia, and neoplastic epithelial foci comparable to the ones observed in WT and TgRANKL mice (Figure 3F,G).

The expression level of total *Rankl* in tumors was higher in genotypes expressing *huRankl* compared to WT mice, whereas no significant differences were identified for the expression levels of *Rank*, *Opg*, *Pr*, and *Erα* among the three genotypes (Figure 3H). Quantification of the phosphorylated proteins downstream of the RANK signaling cascade showed similar levels for p-AKT1, p-STAT3, p-SMAD3, p-ERK1, p-MEK1, and p-EGFR in the tumors from all genotypes, confirming also at the molecular level that the humanized mice respond to MPA/DMPA-induced carcinogenesis similarly to WT and TgRANKL mice (Figure 3I).

### 3.4. Prophylactic Denosumab Treatment Attenuates the Development of MPA/DMBA-Induced Mammary Carcinogenesis in HumTgRANKL Mice

**Figure 4:**
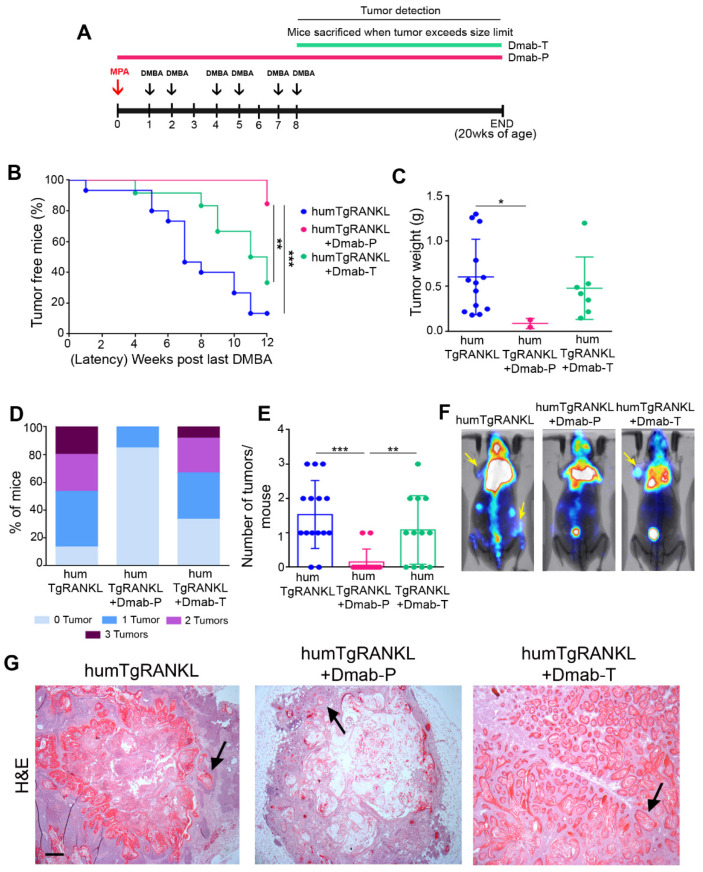
Dmab attenuates MPA/DMBA-induced mammary tumor formation in humTgRANKL mice. (**A**) Carcinogenesis scheme by MPA/DMBA in humTgRANKL mice. Dmab was administered at 10 mg/kg subcutaneously twice weekly, either prophylactically (Dmab-P) or therapeutically (Dmab-T). (**B**) Onset kinetics of MPA/DMBA-induced mammary tumors in humTgRANKL mice treated with Dmab. (**C**) Tumor weight, (**D**) % of mice bearing from 0 to 3 tumors per experimental group, and (**E**) number of tumors per mouse in each experimental group. Data are shown as mean ± SD. Comparison was performed with οne-way ANOVA (* *p* < 0.05, ** *p* < 0.01, *** *p* < 0.001). (**F**) Representative images from planar PET scanning. Yellow arrows indicate tumor signal. (**G**) Representative sections of MPA/DMBA-induced tumors stained with hematoxylin/eosin (n = 2–8/group). Black arrows show keratin pearls. Scale bar: 100 μm.

To examine whether the tumors developed in humTgRANKL mice were huRANKL-dependent, we treated such mice with Denosumab (Dmab), a human monoclonal IgG2 antibody that binds huRANKL with high affinity, preventing its interaction with the RANK receptor. Dmab was administered in humTgRANKL mice either prophylactically before MPA/DMBA treatment (Dmab-P) or therapeutically after the last dose of DMBA (Dmab-T), while control humanized mice were left untreated (Figure 4A). The efficacy of Dmab treatment was assessed through the microCT analysis of femurs, revealing that the prophylactic treatment prevented both trabecular and cortical bone loss, while the therapeutic treatment was less effective as it restored only the cortical bone structure (Figure S5).

In MPA/DMBA-treated humTgRANKL mice, prophylactic treatment with Dmab significantly attenuated the occurrence of mammary tumors (15.4% incidence) in comparison to untreated mice (86.7% incidence). More specifically, from the thirteen mice of the Dmab-P group, only two developed small-size tumors in a single mammary gland throughout the whole duration of the study (Figure 4B–E). The therapeutic treatment of humTgRANKL mice with Dmab did not lead to any significant attenuation of the tumor incidence and tumor burden compared to control mice (Figure 4B–E). Planar PET scanning detected mammary tumors in the untreated and Dmab-T groups (Figure 4F), which histologically had similar characteristics, exhibiting the aberrant formation of keratin pearls. Instead, the tumors developed in the Dmab-P group had limited sizes, with sparse keratin foci (Figure 4G).

**Figure 5:**
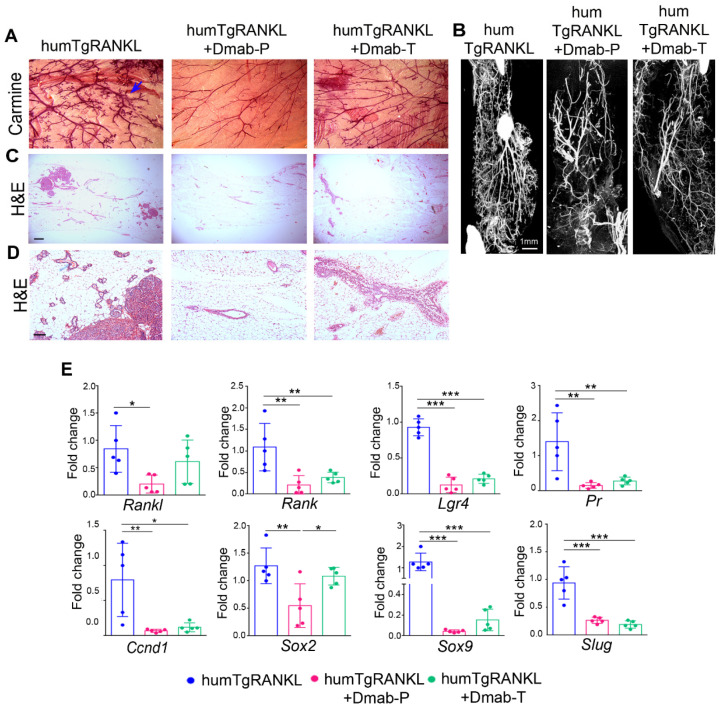
Dmab restores mammary gland density and prevents neoplastic lesions in MPA/DMBA-induced carcinogenesis in humTgRANKL mice. Mammary gland density was evaluated in (**A**) whole-mounts stained with carmine alum and (**B**) reconstructed PTA-enhanced microCT images of the mammary gland ductal tree. (**C**,**D**) Mammary sections stained with hematoxylin/eosin (Scale bar, 150 μm for (**C**) and 80 μm for (**D**)). (**E**) Relative expression analysis with qPCR for *Rankl*, *Rank*, *Lgr4*, *Pr*, *Cyclind1* (*Ccnd1*), *Sox2*, *Sox9* and *Slug* in abdominal mammary glands from MPA/DMBA-treated humTgRANKL mice in the presence or absence of Dmab treatment (n = 5/group). Data are shown as mean ± SD. One-way ANOVA was performed for statistical analysis (* *p* < 0.05, ** *p* < 0.01, *** *p* < 0.001). Blue arrow indicates mammary hyperplastic foci.

We then examined the mammary glands of MPA/DMBA-treated mice to investigate the effects of Dmab in early carcinogenesis stages. Whole-mount carmine staining (Figure 5A) as well as measurements of ductal parameters in 3D microCT images (Figure 5B and Figure S6) showed that increased mammary density, ductal side-branching, and neoplastic areas were prevented by Dmab treatment. Histological analysis of mammary gland sections showed that prophylactic Dmab treatment fully restored normal mammary density and prevented epithelial hyperplasia and neoplasia, while the therapeutic administration of Dmab also prevented neoplasia, but not ductal hyperplasia (Figure 5C,D).

Molecular analysis confirmed the histological findings in mammary glands, showing decreased expressions of *Rankl*, *Rank*, *Lgr4*, *Pr* and *Ccnd1,* while the mammary stem cell markers *Sox2*, *Sox9,* and *Slug* were also significantly decreased in Dmab-treated groups compared to control humTgRANKL mice (Figure 5E). These results indicate that the MPA/DMBA-induced tumors in humTgRANKL mice were dependent on huRANKL, and prophylactic Dmab treatment was superior to therapeutic since it resulted in a markedly delayed onset and decreased tumor incidence and progression.

## 4. Discussion

RANKL is implicated in breast physiology, mediating the paracrine effects of progesterone in the expansion of mammary progenitor cells during the estrous cycle and pregnancy. Moreover, RANKL is also critical in the development of hormone-induced (MPA/DMBA) mammary carcinogenesis in mice [17,19]. It has already been shown that MPA promotes the expression of RANKL in the mammary epithelial cells, preventing apoptosis induced by the DNA-damaging agent DBMA. The pro-survival effect of RANKL/RANK signaling in the damaged epithelium is proposed to cause mammary carcinogenesis in the MPA/DMBA model [17]. Apart from its cognate receptor RANK, RANKL also interacts with the leucine-rich repeat containing G protein-coupled receptor 4 (LGR4) that stimulates breast cancer initiation and metastasis through Wnt signaling [33]. Such evidence provided a link between RANKL and breast carcinogenesis, suggesting RANKL as a target for the prevention of breast cancer. Therefore, the blockade of RANKL via Dmab could inhibit the promitogenic role of progesterone, rationalizing its use as a candidate for drug repurposing in oncology, including breast cancer, beyond skeletal-related event (SREs) prevention.

However, the clinical relevance of RANKL as a target in breast cancer remains enigmatic. In a large population-based cohort study of postmenopausal women with a history of oral bisphosphonate exposure, the use of Dmab was associated with a modestly significant 13% decreased risk of subsequent breast cancer, suggesting a potential protective effect of Dmab use on breast cancer risk in older women previously treated with bisphosphonates [34]. Accumulating evidence points to the amplification of progesterone responsiveness in precancerous tissue from BRCA1 mutation carriers that is mediated by RANKL signaling promoting the expansion of RANK-positive mammary progenitor cells [35,36]. The genetic deletion of RANK in the mammary epithelium or the inhibition of RANKL in *Brca1*-deficient mice substantially delayed hyperplasia and tumor onset [35,36]. Thus, Dmab is currently evaluated in the BRCA-P trial as a preventive option in healthy *BRCA1* mutation carriers who have not undergone prophylactic mastectomy [37]. Moreover, the effect of Dmab on early breast cancer has been investigated. Even though data from the ABCSG-18 study revealed that the combinatorial use of Denosumab with standard neoadjuvant therapy (aromatase inhibitor) significantly improved the disease-free survival (DFS) of postmenopausal women with hormone receptor-positive early breast cancer [38], another clinical trial, the D-CARE study, demonstrated that adjuvant Dmab did not improve either bone metastasis-free survival or disease-free survival in women with early breast cancer [39], while bone-related outcomes were improved [40].

Since contradictory data emerge from clinical trials addressing the anticancer properties of Dmab, it cannot yet be endorsed as a standard therapeutic agent against breast cancer [41]. Τhere is, therefore, an imperative need for more preclinical and clinical studies that can further explore the role of Dmab as an anticancer treatment in either a prophylactic or a therapeutic manner.

In the current study, we investigated whether the expression of *huRankl* in the mammary glands of transgenic mice (TgRANKL) affects their susceptibility to MPA/DMBA-induced mammary carcinogenesis. Even though we would expect higher susceptibility in TgRANKL mice, our results demonstrate that TgRANKL mice overexpressing huRANKL in the mammary glands show similar susceptibility to MPA/DBMA-induced mammary carcinogenesis to WT mice. The prevalent tumor type was adenosquamous carcinoma, possibly derived from squamous metaplasia, which is frequently observed in chemical-induced carcinogenesis in mice [29]. Since both genotypes express the endogenous muRANKL, we could speculate that its levels are sufficient to induce carcinogenesis through RANK signaling. Given that the expression levels of the *Rank* gene are similarly upregulated in the mammary glands of WT and TgRANKL mice upon treatment with MPA/DMBA, it is possible that the RANK levels are the limiting factor that determines disease burden, even when RANKL is overexpressed in TgRANKL mice. Indeed, the genetic modulation of RANK levels affected the MPA/DMBA-driven carcinogenesis, as shown by the mammary-specific RANK deletion that markedly delayed the onset of tumors, while RANK overexpression in the mammary gland increased susceptibility in mammary tumors [17,19]. Nevertheless, as shown in our results, the prophylactic treatment of WT and TgRANKL mice with OPG-Fc, a dual inhibitor of mouse and human RANKL, led to a reduction in the tumor burden both in WT and TgRANKL mice, restored mammary ductal density and prevented the formation of ductal branching and neoplastic foci in adjacent mammary glands, indicating that the MPA/DMBA-induced tumors are RANKL-dependent. However, once formed, the tumors had similar weights and characteristics compared to mice that had not received OPG-Fc treatment, thus raising concerns over the efficacy of OPG-Fc treatment in preventing MPA/DMBA-induced mammary carcinogenesis.

It is well known that the preclinical evaluation of Dmab is unfeasible in mice because, due to species specificity, it cannot bind to endogenous muRANKL. To investigate the exclusive effect of huRANKL in hormone-induced mammary carcinogenesis and to develop a preclinical tool for Dmab evaluation, we generated a humanized transgenic mouse model (humTgRANKL) expressing only huRANKL by breeding the TgRANKL mice with the RANKL^tles/tles^ mice carrying a functional mutation in the endogenous *muRankl* gene [21]. Humanized mice developed tumors with similar latency and burden compared to WT and TgRANKL mice. To examine whether the tumors developed in humTgRANKL mice were huRANKL-dependent, we treated such mice with Dmab, either prophylactically or therapeutically. Prophylactic treatment with Dmab significantly attenuated the latency of mammary tumors and prevented the early stages of carcinogenesis, including hyperplasia formation and preneoplastic lesions. However, the therapeutic treatment of humanized mice with Dmab did not lead to any significant attenuation of tumor incidence or the tumor burden compared to control mice. Therefore, humTgRANKL mice constitute a unique genetic system to preclinically evaluate novel drugs targeting RANKL-mediated signaling.

RANKL stimulates mammary cells through its binding to RANK and LGR4 receptors, which are expressed in breast cancer and are implicated in mammary pathophysiology [33,42]. Herein, we have demonstrated that the expression levels of genes encoding RANK and LGR4 were significantly reduced in mammary glands of MPA/DMBA-treated humanized mice when they received Dmab, either prophylactically or therapeutically. The blockade of the expression of these receptors in the mammary epithelial cells could prevent downstream proliferative signaling. In mammary epithelial cells, the cyclin D1 pathway is an essential signaling pathway activated downstream of RANKL/RANK interaction that promotes mammary cell proliferation, expansion, and survival [43]. The treatment of humTgRANKL mice with Dmab decreased the expression levels of the *ccnd1* gene encoding cyclin D1, supporting the antiproliferative effects of Dmab in the mammary gland during MPA/DMBA-induced carcinogenesis. To investigate further the signaling cascade activated downstream of RANKL, we developed a multiplex for phosphoproteins, and detected them in mammary glands and tumors. We identified an increased presence of phosphoproteins such as p-AKT1, p-STAT3, p-SMAD3, p-CREB1, p-p38, p-PTN11, and p-ERK1 in mammary tumors compared to the mammary gland, which should be further investigated as potential novel biomarkers in mammary carcinogenesis.

Since progesterone is essential for the maintenance and expansion of mammary gland stem cells (MaSCs), which could serve as a target for transformation and progression to breast carcinogenesis [12], we investigated whether the blockade of RANKL could result in the loss of mammary cells expressing stem cell markers. Indeed, MaSC markers such as Sox2, Sox9 and Slug were significantly reduced in adjacent mammary glands of humanized mice treated with Dmab either prophylactically or therapeutically, highlighting the importance of RANKL signaling in the expansion of MaSCs during hormone-induced carcinogenesis.

## 5. Conclusions

In conclusion, by developing a humanized transgenic mouse model of huRANKL overexpression, we demonstrated that huRANKL mediated the MPA/DMBA-induced mammary carcinogenesis, while its prophylactic inhibition by Dmab prevented tumorigenesis. Since the effect of Dmab in breast cancer is promising for BRCA mutation carriers and a subgroup of patients in early breast cancer, though inconsistently [38,39,44], our humTgRANKL mice provide a unique genetic tool for investigating the involvement of huRANKL as a driver gene in breast cancer pathogenesis, and can serve as a preclinical platform for anticancer therapies. 

## Data Availability

The data presented in this study are available on request from the corresponding author.

## Supplementary Materials

The following supporting information can be downloaded at: https://www.mdpi.com/article/10.3390/cancers15154006/s1, Figure S1: Histological analysis of MPA/DMBA-induced mammary tumors in WT and TgRANKL mice; Figure S2: Additional multiplex ELISA analysis for phospho-proteins; Figure S3: OPG-Fc treatment prevented bone resorption in WT and TgRANKL mice; Figure S4: Humanized TgRANKL mice developed an osteoporotic phenotype similar to TgRANKL mice; Figure S5: Effect of either prophylactic or therapeutic treatment of humTgRANKL mice with denosumab in bone architecture; Figure S6: Denosumab restores MPA/DMBA-induced mammary gland density in humTgRANKL mice; Table S1: Primer sequences used in qPCR; Table S2: Protein assays of the custom-developed 19-plex assay panel and their phosphorylation residues.

## References

[1] Sigl V., Jones L.P., Penninger J.M. (2016). RANKL/RANK: From Bone Loss to the Prevention of Breast Cancer. Open Biol..

[2] Henderson B.E., Ross R.K., Judd H.L., Krailo M.D., Pike M.C. (1985). Do Regular Ovulatory Cycles Increase Breast Cancer Risk?. Cancer.

[3] Söderqvist G. (1998). Effects of Sex Steroids on Proliferation in Normal Mammary Tissue. Ann. Med..

[4] Banks E., Beral V., Bull D., Reeves G., Austoker J., English R., Patnick J., Peto R., Vessey M., Wallis M. (2003). Breast Cancer and Hormone-Replacement Therapy in the Million Women Study. Lancet.

[5] Beverly R., Volkar J. (2021). Risks and Benefits of Estrogen Plus Progestin in Healthy Postmenopausal Women. 50 Studies Every Obstetrician-Gynecologist Should Know.

[6] Olver I.N. (2016). Prevention of Breast Cancer. Med. J. Aust..

[7] Narod S.A. (2011). Hormone Replacement Therapy and the Risk of Breast Cancer. Nat. Rev. Clin. Oncol..

[8] Fuller K., Wong B., Fox S., Choi Y., Chambers T.J. (1998). TRANCE Is Necessary and Sufficient for Osteoblast-Mediated Activation of Bone Resorption in Osteoclasts. J. Exp. Med..

[9] Takahashi N., Udagawa N., Suda T. (1999). A New Member of Tumor Necrosis Factor Ligand Family, ODF/OPGL/TRANCE/RANKL, Regulates Osteoclast Differentiation and Function. Biochem. Biophys. Res. Commun..

[10] Anandarajah A.P. (2009). Role of RANKL in Bone Diseases. Trends Endocrinol. Metab..

[11] Silva-Fernández L., Rosario M.P., Martínez-López J.A., Carmona L., Loza E. (2013). Denosumab for the Treatment of Osteoporosis: A Systematic Literature Review. Reumatol. Clin..

[12] Joshi P.A., Jackson H.W., Beristain A.G., Di Grappa M.A., Mote P.A., Clarke C.L., Stingl J., Waterhouse P.D., Khokha R. (2010). Progesterone Induces Adult Mammary Stem Cell Expansion. Nature.

[13] Asselin-Labat M.-L., Vaillant F., Sheridan J.M., Pal B., Wu D., Simpson E.R., Yasuda H., Smyth G.K., Martin T.J., Lindeman G.J. (2010). Control of Mammary Stem Cell Function by Steroid Hormone Signalling. Nature.

[14] Fernandez-Valdivia R., Lydon J.P. (2012). From the Ranks of Mammary Progesterone Mediators, RANKL Takes the Spotlight. Mol. Cell. Endocrinol..

[15] Fata J.E., Kong Y.Y., Li J., Sasaki T., Irie-Sasaki J., Moorehead R.A., Elliott R., Scully S., Voura E.B., Lacey D.L. (2000). The Osteoclast Differentiation Factor Osteoprotegerin-Ligand Is Essential for Mammary Gland Development. Cell.

[16] Van Poznak C., Cross S.S., Saggese M., Hudis C., Panageas K.S., Norton L., Coleman R.E., Holen I. (2006). Expression of Osteoprotegerin (OPG), TNF Related Apoptosis Inducing Ligand (TRAIL), and Receptor Activator of Nuclear Factor ΚB Ligand (RANKL) in Human Breast Tumours. J. Clin. Pathol..

[17] Schramek D., Leibbrandt A., Sigl V., Kenner L., Pospisilik J.A., Lee H.J., Hanada R., Joshi P.A., Aliprantis A., Glimcher L. (2010). Osteoclast Differentiation Factor RANKL Controls Development of Progestin-Driven Mammary Cancer. Nature.

[18] Infante M., Fabi A., Cognetti F., Gorini S., Caprio M., Fabbri A. (2019). RANKL/RANK/OPG System beyond Bone Remodeling: Involvement in Breast Cancer and Clinical Perspectives. J. Exp. Clin. Cancer Res..

[19] Gonzalez-Suarez E., Jacob A.P., Jones J., Miller R., Roudier-Meyer M.P., Erwert R., Pinkas J., Branstetter D., Dougall W.C. (2010). RANK Ligand Mediates Progestin-Induced Mammary Epithelial Proliferation and Carcinogenesis. Nature.

[20] Rinotas V., Niti A., Dacquin R., Bonnet N., Stolina M., Han C.-Y., Kostenuik P., Jurdic P., Ferrari S., Douni E. (2014). Novel Genetic Models of Osteoporosis by Overexpression of Human RANKL in Transgenic Mice. J. Bone Miner. Res..

[21] Douni E., Rinotas V., Makrinou E., Zwerina J., Penninger J.M., Eliopoulos E., Schett G., Kollias G. (2012). A RANKL G278R Mutation Causing Osteopetrosis Identifies a Functional Amino Acid Essential for Trimer Assembly in RANKL and TNF. Hum. Mol. Genet..

[22] Kelloff G.J., Hoffman J.M., Johnson B., Scher H.I., Siegel B.A., Cheng E.Y., Cheson B.D., O’Shaughnessy J., Guyton K.Z., Mankoff D.A. (2005). Progress and Promise of FDG-PET Imaging for Cancer Patient Management and Oncologic Drug Development. Clin. Cancer Res..

[23] Nanni C., Pettinato C., Ambrosini V., Spinelli A., Trespidi S., Rubello D., Al-Nahhas A., Franchi R., Alavi A., Fanti S. (2007). Retro-Orbital Injection Is an Effective Route for Radiopharmaceutical Administration in Mice during Small-Animal PET Studies. Nucl. Med. Commun..

[24] Kolokotroni A., Gkikopoulou E., Rinotas V., Douni E. (2023). Phosphotungstic Acid-Enhanced Microcomputed Tomography for Quantitative Visualization of Mouse Mammary Gland Morphology. J. Med. Imaging.

[25] Bouxsein M.L., Boyd S.K., Christiansen B.A., Guldberg R.E., Jepsen K.J., Müller R. (2010). Guidelines for Assessment of Bone Microstructure in Rodents Using Micro-Computed Tomography. J. Bone Miner. Res..

[26] Li G.-W., Chang S.-X., Fan J.-Z., Tian Y.-N., Xu Z., He Y.-M. (2013). Marrow Adiposity Recovery after Early Zoledronic Acid Treatment of Glucocorticoid-Induced Bone Loss in Rabbits Assessed by Magnetic Resonance Spectroscopy. Bone.

[27] Poussin C., Mathis C., Alexopoulos L.G., Messinis D.E., Dulize R.H.J., Belcastro V., Melas I.N., Sakellaropoulos T., Rhrissorrakrai K., Bilal E. (2014). The Species Translation Challenge—A Systems Biology Perspective on Human and Rat Bronchial Epithelial Cells. Sci. Data.

[28] Hu H., Wang J., Gupta A., Shidfar A., Branstetter D., Lee O., Ivancic D., Sullivan M., Chatterton R.T., Dougall W.C. (2014). RANKL Expression in Normal and Malignant Breast Tissue Responds to Progesterone and Is Up-Regulated during the Luteal Phase. Breast Cancer Res. Treat..

[29] Cardiff R.D., Anver M.R., Gusterson B.A., Hennighausen L., Jensen R.A., Merino M.J., Rehm S., Russo J., Tavassoli F.A., Wakefield L.M. (2000). The Mammary Pathology of Genetically Engineered Mice: The Consensus Report and Recommendations from the Annapolis Meeting. Oncogene.

[30] Miller R.E., Branstetter D., Armstrong A., Kennedy B., Jones J., Cowan L., Bussiere J., Dougall W.C. (2007). Receptor Activator of NF-ΚB Ligand Inhibition Suppresses Bone Resorption and Hypercalcemia but Does Not Affect Host Immune Responses to Influenza Infection. J. Immunol..

[31] Ominsky M.S., Kostenuik P.J., Cranmer P., Smith S.Y., Atkinson J.E. (2007). The RANKL Inhibitor OPG-Fc Increases Cortical and Trabecular Bone Mass in Young Gonad-Intact Cynomolgus Monkeys. Osteoporos. Int..

[32] Ominsky M.S., Li X., Asuncion F.J., Barrero M., Warmington K.S., Dwyer D., Stolina M., Geng Z., Grisanti M., Tan H.L. (2008). RANKL Inhibition with Osteoprotegerin Increases Bone Strength by Improving Cortical and Trabecular Bone Architecture in Ovariectomized Rats. J. Bone Miner. Res..

[33] Yue Z., Yuan Z., Zeng L., Wang Y., Lai L., Li J., Sun P., Xue X., Qi J., Yang Z. (2018). LGR4 Modulates Breast Cancer Initiation, Metastasis, and Cancer Stem Cells. FASEB J..

[34] Giannakeas V., Cadarette S.M., Ban J.K., Lipscombe L., Narod S.A., Kotsopoulos J. (2018). Denosumab and Breast Cancer Risk in Postmenopausal Women: A Population-Based Cohort Study. Br. J. Cancer.

[35] Sigl V., Owusu-Boaitey K., Joshi P.A., Kavirayani A., Wirnsberger G., Novatchkova M., Kozieradzki I., Schramek D., Edokobi N., Hersl J. (2016). RANKL/RANK Control Brca1 Mutation-Driven Mammary Tumors. Cell Res..

[36] Nolan E., Vaillant F., Branstetter D., Pal B., Giner G., Whitehead L., Lok S.W., Mann G.B., Rohrbach K., Huang L.Y. (2016). RANK Ligand as a Potential Target for Breast Cancer Prevention in BRCA1-Mutation Carriers. Nat. Med..

[37] Singer C.F. (2021). Nonsurgical Prevention Strategies in BRCA1 and BRCA2 Mutation Carriers. Breast Care.

[38] Gnant M., Pfeiler G., Steger G.G., Egle D., Greil R., Fitzal F., Wette V., Balic M., Haslbauer F., Melbinger-Zeinitzer E. (2019). Adjuvant Denosumab in Postmenopausal Patients with Hormone Receptor-Positive Breast Cancer (ABCSG-18): Disease-Free Survival Results from a Randomised, Double-Blind, Placebo-Controlled, Phase 3 Trial. Lancet Oncol..

[39] Coleman R., Finkelstein D.M., Barrios C., Martin M., Iwata H., Hegg R., Glaspy J., Periañez A.M., Tonkin K., Deleu I. (2020). Adjuvant Denosumab in Early Breast Cancer (D-CARE): An International, Multicentre, Randomised, Controlled, Phase 3 Trial. Lancet Oncol..

[40] Coleman R., Zhou Y., Jandial D., Cadieux B., Chan A. (2021). Bone Health Outcomes from the International, Multicenter, Randomized, Phase 3, Placebo-Controlled D-CARE Study Assessing Adjuvant Denosumab in Early Breast Cancer. Adv. Ther..

[41] Deligiorgi M.V., Panayiotidis M.I., Trafalis D.T. (2020). Repurposing Denosumab in Breast Cancer beyond Prevention of Skeletal Related Events: Could Nonclinical Data Be Translated into Clinical Practice?. Expert Rev. Clin. Pharmacol..

[42] Wu X., Li F., Dang L., Liang C., Lu A., Zhang G. (2020). RANKL/RANK System-Based Mechanism for Breast Cancer Bone Metastasis and Related Therapeutic Strategies. Front. Cell Dev. Biol..

[43] Cao Y., Bonizzi G., Seagroves T.N., Greten F.R., Johnson R., Schmidt E.V., Karin M. (2001). IKKα Provides an Essential Link between RANK Signaling and Cyclin D1 Expression during Mammary Gland Development. Cell.

[44] Ciscar M., Trinidad E.M., Perez-Montoyo H., Alsaleem M., Jimenez-Santos M.J., Toss M., Sanz-Moreno A., Vethencourt A., Perez-Chacon G., Petit A. (2023). RANK Is an Independent Biomarker of Poor Prognosis in Estrogen Receptor-Negative Breast Cancer and a Therapeutic Target in Patient-Derived Xenografts. EMBO Mol. Med..

