# Supplementary material for: Α Humanized RANKL Transgenic Mouse Model of Progestin-Induced Mammary Carcinogenesis for Evaluation of Novel Therapeutics
Source: Cancers (Basel). 2023 Aug 7;15(15):4006. doi: 10.3390/cancers15154006 (PMC10417415; doi:10.3390/cancers15154006)
Supplement: Supplementary file 1 [file cancers-15-04006-s001.zip › cancers-2535425-supplementary.pdf]

Supplementary materials

A humanized RANKL transgenic mouse model of progestin-induced mammary carcinogenesis for evaluation of novel therapeutics

Anthi Kolokotroni[1,2], Evi Gkikopoulou[1,2], Vagelis Rinotas[2], Lydia Ntari[3], Danae Zareifi[4], Maritina Rouchota[5], Sophia Sarpaki[5], Ilias Lymperopoulos[6], Leonidas G Alexopoulos[4], George Loudos[5], Maria C Denis[3], Niki Karagianni[3], Eleni Douni[1,2,*]

[1]Laboratory of Genetics, Department of Biotechnology, Agricultural University of Athens, Iera Odos 75, 11855 Athens, Greece

[2]Institute for Bioinnovation, Biomedical Sciences Research Center "Alexander Fleming", Fleming 34, 16672 Vari, Greece

[3]Biomedcode Hellas SA, Fleming 34, 16672 Vari, Greece

[4]Department of Mechanical Engineering, National Technical University of Athens, 10682 Athens, Greece

[5]BIOEMTECH, Lefkippos Attica Technology Park, NCSR “Demokritos”, 15343 Ag. Paraskevi-Athens, Greece

[6]1rst Breast Clinic, Iaso Hospital, 37-39 Kifissias, 15123 Marousi, Greece

***** Correspondence: douni@aua.gr or douni@fleming.gr

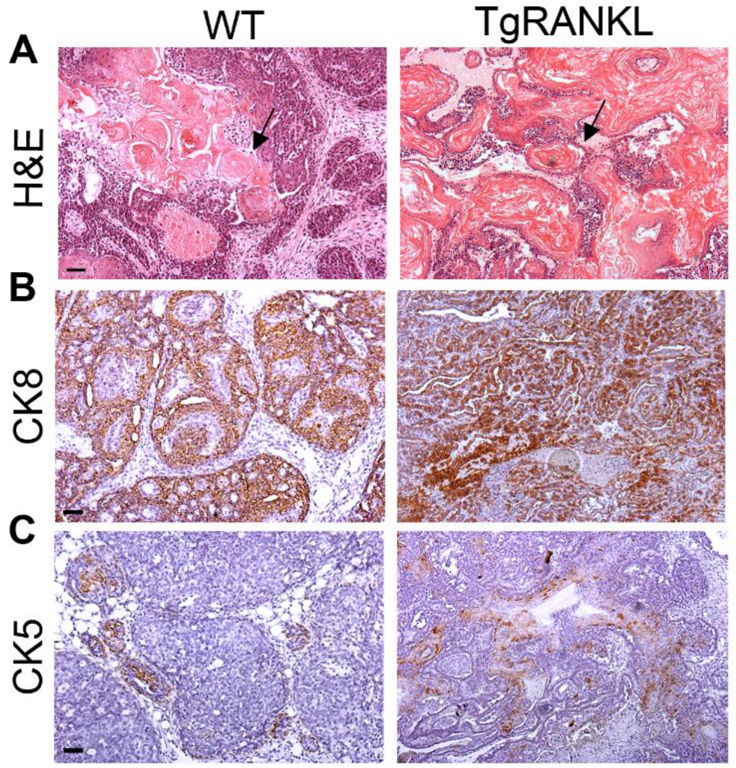

Figure S1. Histological analysis of MPA/DMBA-induced mammary tumors in WT and TgRANKL mice. Representative histological sections of mammary tumors isolated from WT and TgRANKL mice stained with **(A)** hematoxylin and eosin, **(B)** Cytokeratin 8, and **(C)** Cytokeratin 5 (n=3/group). Keratin pearls are indicated by arrows. Scale bar, 80µm.

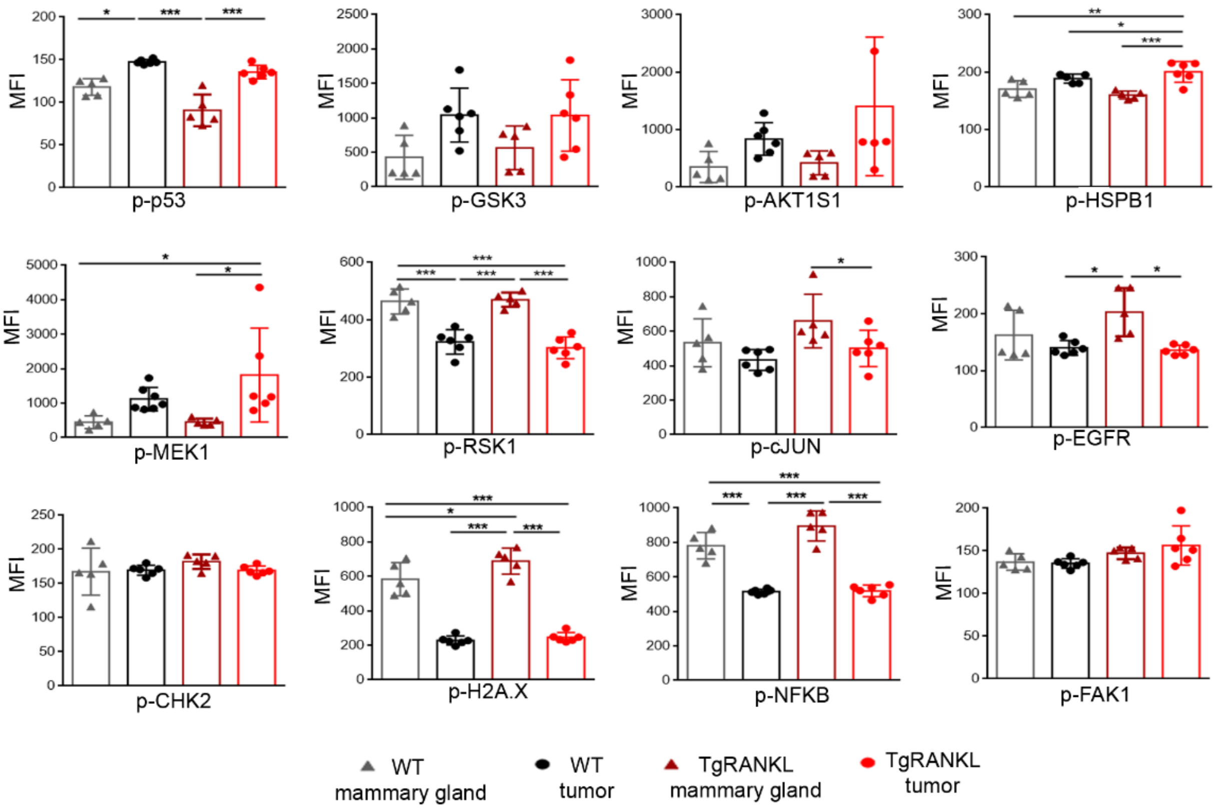

Figure S2. Additional multiplex ELISA analysis for phospho-proteins. p-p53, p-GSK3, p-AKT1S1, p-HSPB1, p-MEK1, p-RSK1, p-cJUN, p-EGFR, p-CHK2, p-H2A.X, p-NFκB and p-FAK1 were measured in mammary glands and tumors from WT and TgRANKL mice (n=5=6/group). Comparison was performed with one-way ANOVA and Tukey's post hoc test (* $p<0.05$, ** $p<0.01$, *** $p<0.001$).

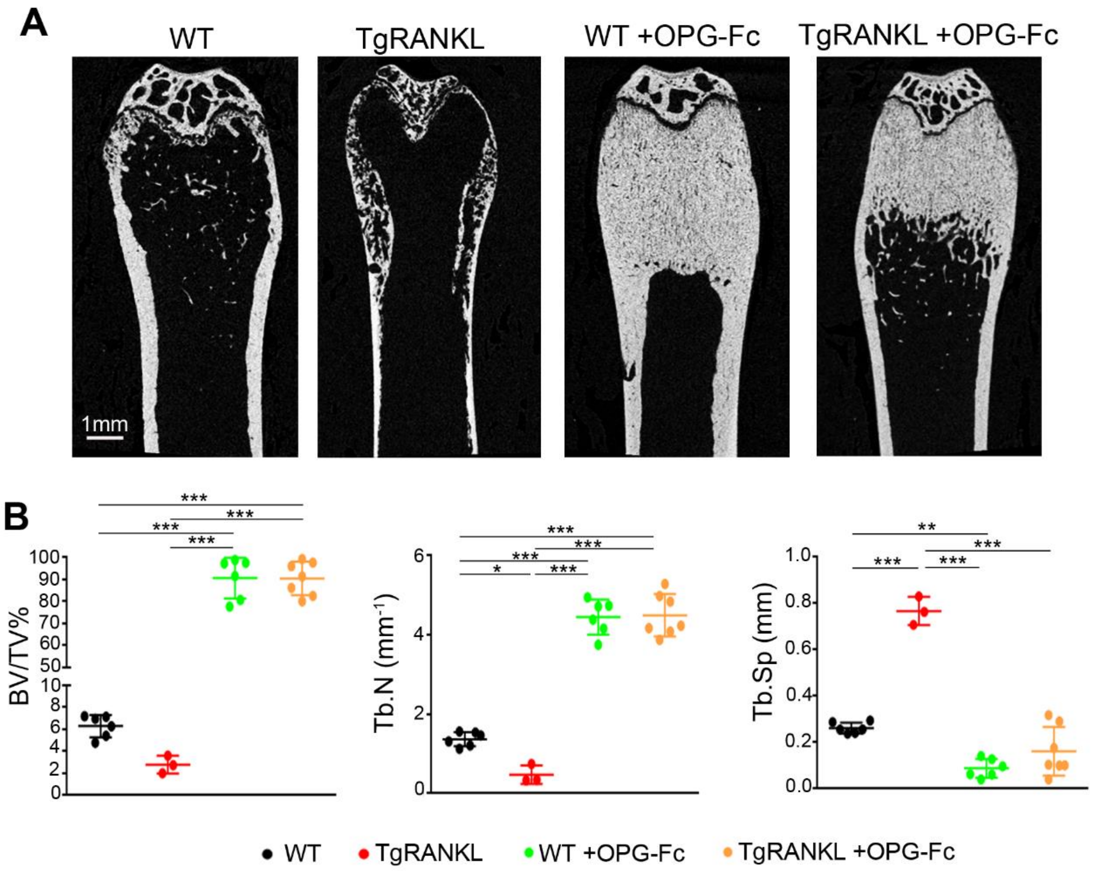

Figure S3. OPG-Fc treatment prevented bone resorption in WT and TgRANKL mice. (A) Representative microCT 2D images and **(B)** quantitative analysis for BV/TV%, Tb.N, and Tb.Sp in the trabecular bone of femurs from MPA/DMBA-treated WT and TgRANKL mice with or without OPG-Fc treatment (n = 3-7/group). Data are shown as mean ± SD. One-Way ANOVA was performed for statistical analysis (*p < 0.05, **p <0.01, ***p <0.001).

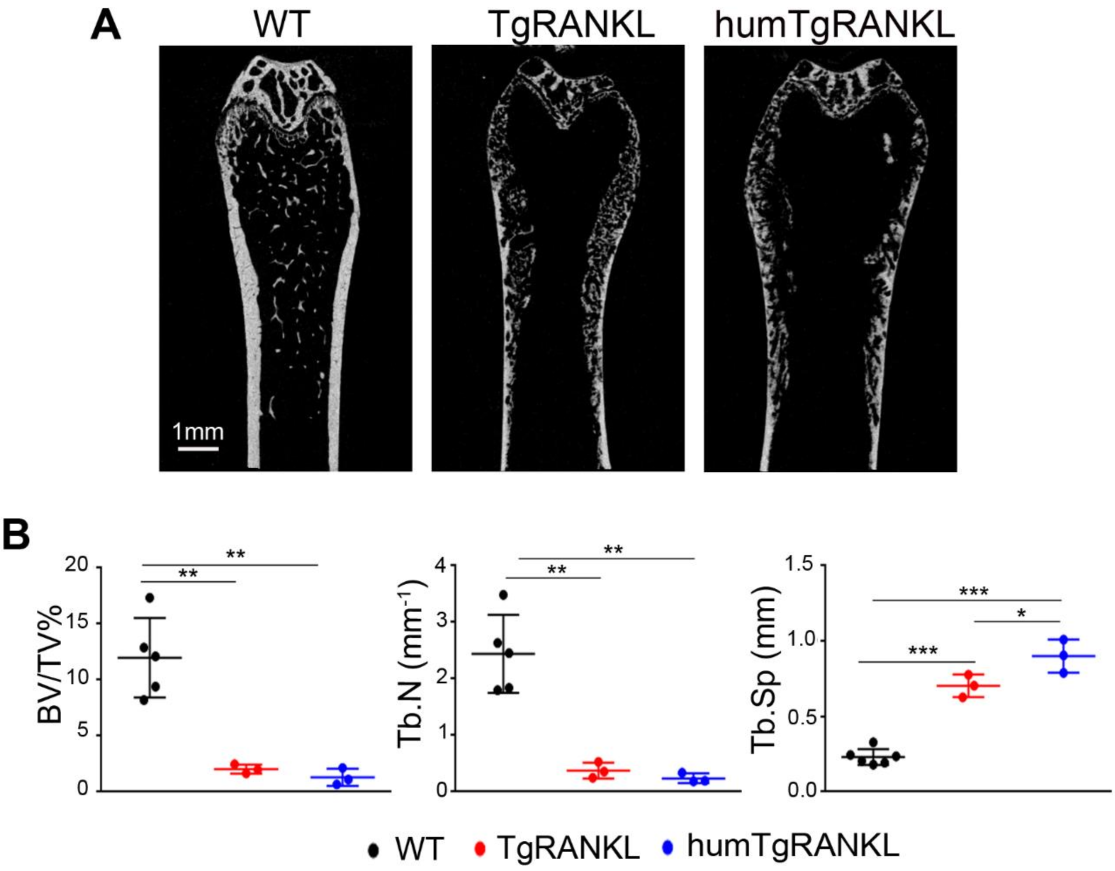

Figure S4. Humanized TgRANKL mice developed an osteoporotic phenotype similar to TgRANKL mice. (A) Representative microCT 2D images and **(B)** quantitative analysis for BV/TV%, Tb.N, and Tb.Sp in the trabecular bone of femurs from WT, TgRANKL and humTgRANKL mice (n = 3-5/ group). Data are shown as mean ± SD. One-Way ANOVA was performed for statistical analysis (*p < 0.05, **p <0.01, ***p <0.001).

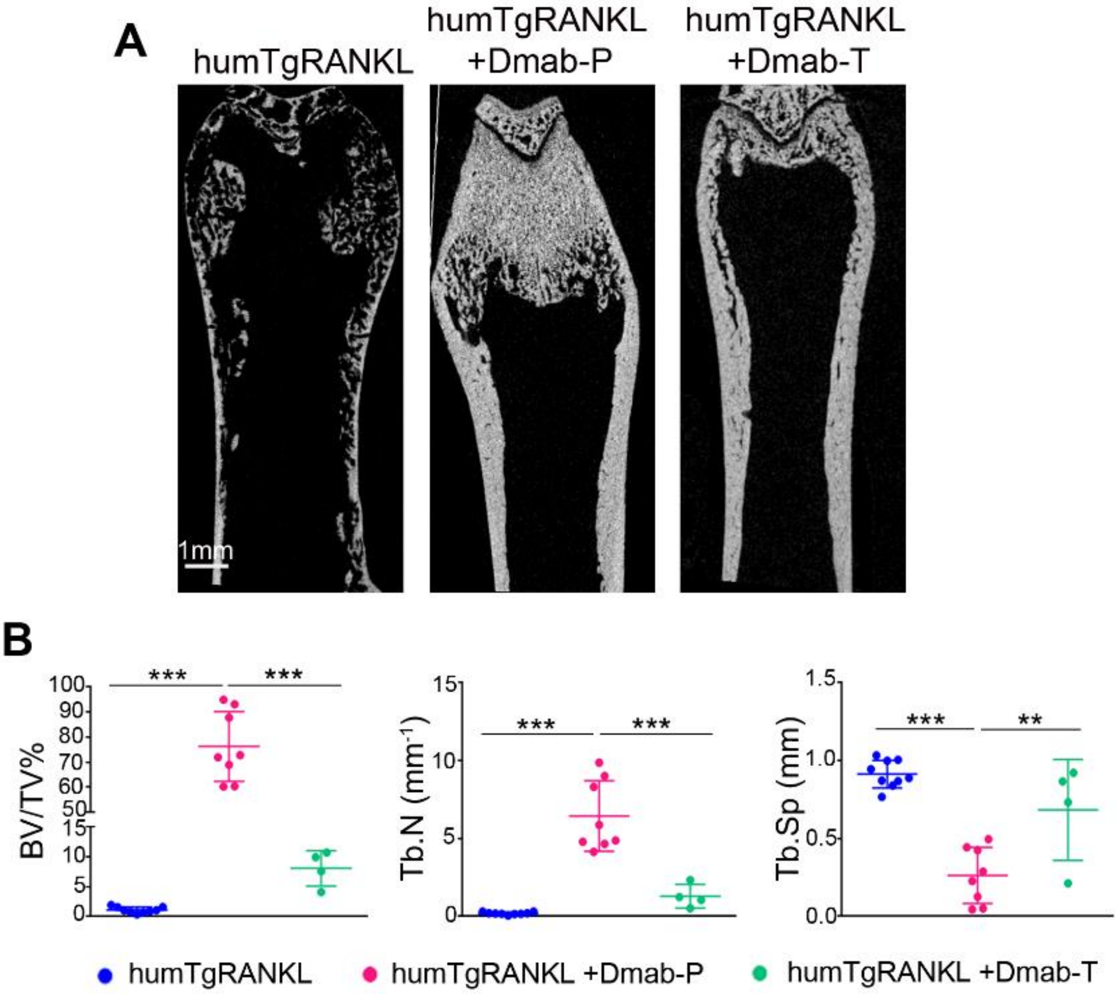

Figure S5. Effect of either prophylactic or therapeutic treatment of humTgRANKL mice with denosumab in bone architecture. (A) Representative microCT 2D images and **(B)** quantitative analysis for BV/TV%, Tb.N, and Tb.Sp in the trabecular bone of femurs from MPA/DMBA-treated humTgRANKL mice without or with Dmab treatment either prophylactically (Dmab-P) or therapeutically (Dmab-T) (n = 4-9 group). Data are shown as mean ± SD. One-Way ANOVA was performed for statistical analysis (**p <0.01, ***p <0.001).

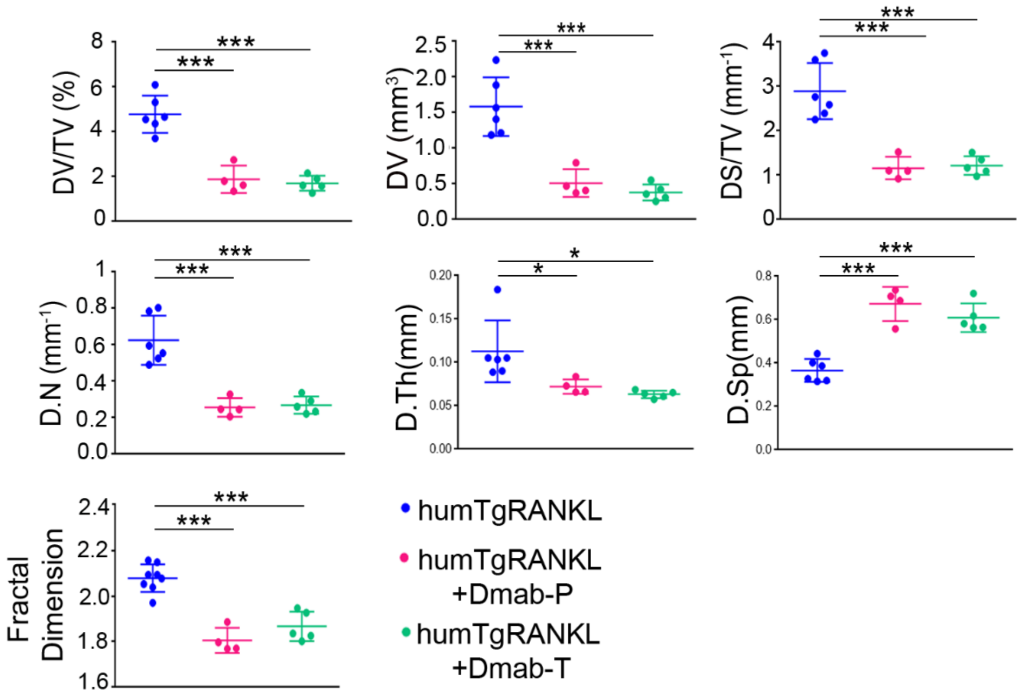

Figure S6. Denosumab restores MPA/DMBA-induced mammary gland density in humTgRANKL mice. Quantitative microCT analysis of mammary gland epithelium from humTgRANKL mice either untreated or treated with Dmab prophylactically or therapeutically (n=4-6 mice/group) DV/TV%: Ductal volume/Tissue volume %, DV (mm^3): Ductal volume, DS/TV (mm^{-1}): Ductal Surface/Tissue Volume, Volume, D.N (mm): Ductal number, D.Th (mm): Ductal Thickness, D.Sp (mm): Ductal Separation, Fractal Dimension. Data are shown as mean ± SD. One-Way ANOVA was performed for statistical analysis (* $p<0.05$, *** $p<0.001$).

Table S1. Primer sequences used in qPCR.

Target genes	Forward (5'-3')	Reverse (5'-3')
B2M	ACATCAAGAAGGTGGTGAAGCAGG	AGTTGCTGTTGAAGTCGCAGGAGA
mu+huRankl	ACCTGTACGCCAACATTTGC	CTTGGG ATTTTGATGCTGGT
huRankl	ACGCGTATTTACAGCCAGTG	5CCCGTAATTGCTCCAATCTG
muRankl	TGTACTTTCGAGCGCAGATG	AGGCTTGTTTCATCCTCCTG
muOpg	TGATGTATGCCCTCAAGCAC	TTGTGAAGCTGTGCAGGAAC
muRank	TCTTATGTTGGGGTCCATCC	AATAAGCTTAGCCCCGAACC
muPr	CTCCGGGACCGAACAGAGT	ACAACAACCCTTTGGTAGCAG
muErα	AATGAAATGGGTGCTTCAGG	ATAGATCATGGGCGGTTCAG
muCcnd1	GCGTACCCTGACACCAATCTC	CTCCTCTTCGCACTTCTGCTC
muLgr5	CCTACTCGAAGACTTACCCAGT	GCATTGGGGTGAATGATAGCA
muLgr4	TACAACTGGCTGGTAACGACC	TTGAGTTCTTTCAACCCAGACAA
muSox2	AAAGGGTTCTTGCTGGGTTT	AGACCACGAAAACGGTCTTG
muSox9	CGGAACAGACTCACATCTCTCC	CTTGCACGTCGGTTTTGG
muSlug	TCAACGCCTCCAAGAAGCCCA	ATAGGGCTGTATGCTCCCGAGGT

Table S2. Protein assays of the custom-developed 19-plex assay panel and their phosphorylation residues.

	**Protein-assay abbreviation**	**Protein name**	**Phosphorylation Residue**
1	Smad3	Mothers against decapentaplegic homolog 3	S423/S425
2	p53	Cellular tumour antigen p53	S15
3	AKTS1	Proline-rich AKT1 substrate 1	T246
4	GSK3	Glycogen synthase kinase-3 alpha/beta	S21/9
5	AKT1	RAC-alpha serine/threonine-protein kinase	S473
6	HSP27 (HSPB1)	Heat shock protein beta-1	S78/S82
7	p38	Mitogen-activated protein kinase 14/11	T180/Y182
8	MEK1	Dual specificity mitogen-activated protein kinase kinase 1	S217/S221
9	RSK1	Ribosomal protein S6 kinase alpha-1	S380
10	CREB1	Cyclic AMP-responsive element-binding protein 1	S133
11	cJUN	Transcription factor Jun	S63
12	EGFR	Epidermal growth factor receptor	Y1068
13	PTN11	Tyrosine-protein phosphatase non-receptor type 11	Y542
14	CHK2	Serine/threonine-protein kinase Chk2	T68
15	H2A.X	Histone H2AX	S139
16	NF-KB	Nuclear factor kappa-light-chain-enhancer of activated B cells	S536
17	STAT3	Signal transducer and activator of transcription 3	Y705
18	ERK1	Mitogen-activated protein kinase 3	T202/Y204
19	FAK1	Focal adhesion kinase 1	Y397
